# Subdural Hemorrhage after Scoliosis and Detethering of Cord Surgery

**DOI:** 10.1155/2018/5061898

**Published:** 2018-04-02

**Authors:** Rohan Bhimani, Fardeen Bhimani, Preeti Singh

**Affiliations:** ^1^Department of Orthopaedics, Hinduja Healthcare Surgicals, 11th Road, Khar (West), Mumbai 400052, India; ^2^Department of Orthopaedics, Bharati Hospital, Pune 411043, India; ^3^Department of Orthopaedics, Osmania General Hospital, Hyderabad 500012, India

## Abstract

**Introduction:**

Intracranial hypotension may occur when CSF leaks from the subarachnoid space. Formation of intracranial, subdural, and subarachnoid hemorrhage has been observed after significant CSF leak as seen in lumbar puncture or ventricular shunt placement. However, very few cases, referring to these remote complications following spine surgery, have been described in literature. We present a case of a 10-year-old male child operated for idiopathic scoliosis with low-lying conus medullaris who postoperatively developed subdural hemorrhage.

**Case Report:**

A case of a 10-year-old male operated for idiopathic scoliosis with low-lying conus medullaris is presented. To correct this, detethering was done at the L3 level, laminectomy was done from L2 to L3 with pedicular screw fixation from T3 to L2, and bone grafting with right costoplasty was done from the 3rd to the 6th ribs. On the 5th day postoperatively, the patient developed convulsions and drowsiness and recovered subsequently by postoperative day 7.

**Conclusion:**

We report a rare case of an acute intracranial subdural hemorrhage caused by intracranial hypotension following scoliosis and detethering of cord surgery. This report highlights the potential morbidity associated with CSF leak occurring after this surgery.

## 1. Introduction

Postoperative subdural hemorrhage following scoliosis and detethering of cord surgery is a disastrous complication whose pathogenesis is unclear in most cases. Very few cases have been recorded in the past with this complication.

We present the case of a 10-year-old male operated for idiopathic scoliosis with low-lying conus medullaris. To correct this, detethering was done at the L3 level, laminectomy was done from L2 to L3 with pedicular screw fixation from T3 to L2, and bone grafting with right costoplasty was done from the 3rd to the 6th ribs. On the 5th day postoperatively, the patient developed convulsions and drowsiness and recovered subsequently by postoperative day 7.

## 2. Case Report

**Figure 1:**
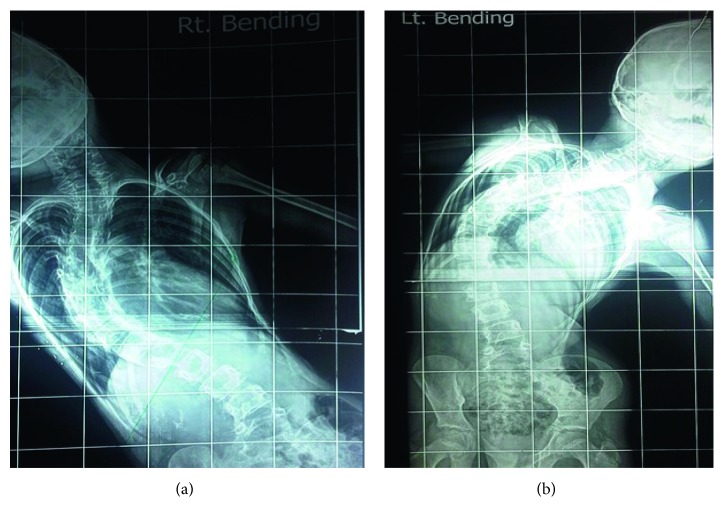
Preoperative X-ray showing right idiopathic thoracolumbar scoliosis.

**Figure 2:**
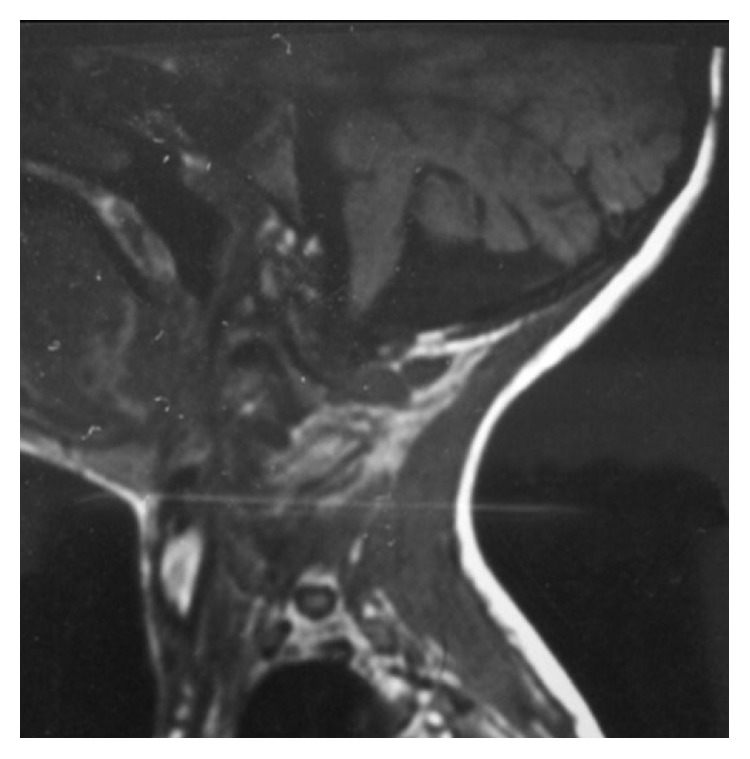
Preoperative MRI brainstem picture showing no abnormalities.

A 10-year-old male presented with complaints of deformity and pain in the back. The preoperative roentogram displayed right idiopathic thoracolumbar scoliosis (Figure 1). The patient was moderately built with no significant neurological disorders. His reports showed haemoglobin 10.3 g/dl, ESR 14 mm/hr, and CRP Q 30 mg/l. PFT values were low suggestive of type II respiratory failure. The patient did not give any history of previous head injury, and his preoperative brainstem magnetic resonance imaging (MRI) did not show any abnormality (Figure 2).

**Figure 3:**
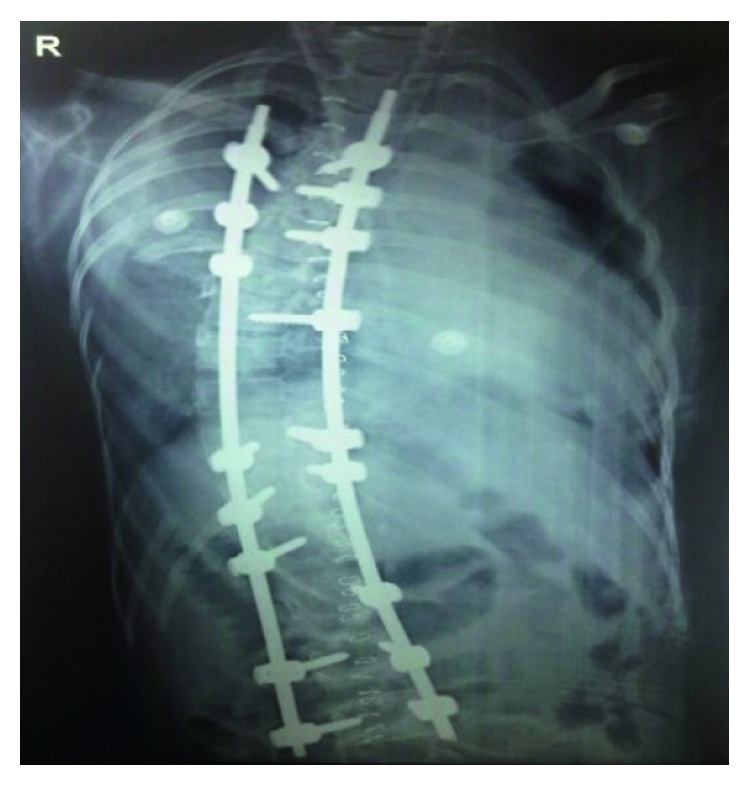
Postoperative X-ray showing correction of scoliosis with implants in situ.

**Figure 4:**
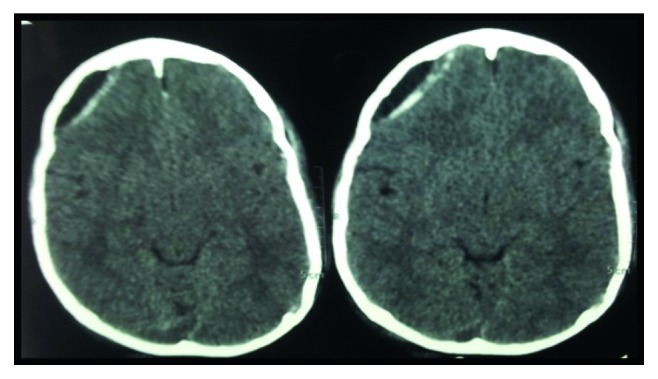
Postoperative CT scan showing subdural hemorrhage in temporoparietal region.

The patient underwent detethering at the L2 level with pedicular screw fixation from T3 to L2 with bone grafting with right costoplasty from the 3rd to the 6th ribs (Figure 3). General anaesthesia was given using Scoline injection (succinylcholine). The patient went into bronchospasm for which theophylline injection and Asthalin injection (salbutamol) were given. An arterial line was put to monitor blood pressure during surgery. He was operated in prone position. Total surgical time was 5 hours with intraoperative blood loss around 1.5 liters. The detethering was performed at the lumbar L2 level, and the dura was repaired. Intraoperatively, there was an arterial bleeder, which led to the fall of BP to 40/20 mmHg because of the rupture of the segmental vessels at T7-T8 of the left side intraoperatively. Hypotension lasted for 5 minutes, which was immediately brought back to normal limits by giving whole blood and colloids. The total CSF loss during the surgery was about 100 ml. Postoperatively, the drain output was 300 ml over 3 days. The patient received 2 units of whole blood and 2 units of packed red cells from the intraoperative period to post-op day 4. Postoperatively, the patient was on a ventilator up to day 7. On post-op day 5, the patient developed convulsions, and CT brain was performed that showed subdural hemorrhage (Figure 4). The neurosurgeon advised conservative management with phenytoin for convulsion. By post-op day 7, the patient recovered completely with no neurological deficit. The patient was discharged 2 weeks after neurosurgery and orthopaedic fitness. The patient was followed up regularly for one year with no other postoperative complications.

## 3. Discussion

Intracranial hypotension associated with subdural hematoma is unusual and can prove to be a devastating complication, but it is a possible complication of any procedure involving the dura. Intracranial hypotension is usually a benign condition; however, it can lead to subdural hemorrhage (SDH) and subarachnoid hemorrhage (SAH) due to laceration of superior cerebral vein or arachnoid granulations [1]. Although the exact mechanism is unknown, it is highly indicative that CSF hypovolemia causes decrease of intracranial pressure, thereby causing enlargement of dural venous sinuses that becomes an important factor in the pathogenesis of subdural hemorrhage. Downward brain displacement resulting from pressure change can lead to transient stretching and occlusion of the previously enlarged venous sinuses and thus dispose the patient to venous tears [2]. This probably is the most likely cause of SDH in our case. There have been few reported cases of acute SDH and SAH after other spinal surgeries. Surgical evacuation of a hematoma was performed in half of these cases. Most of them were associated with an intraoperative dural breach [3–5]. Usually, such tears are readily identified by the surgeons and closed intraoperatively. Sometimes dural tear may go unnoticed or may be prone to rupture later which may result in an occult CSF leak and intracranial hypotension [5–7]. In our case, durotomy done during detethering resulted in significant CSF leak which led to intracranial hypotension, resulting in subdural hemorrhage. Systemic hypotension due to blood loss can lead to intracranial hypotension [8, 9]. In our patient, systemic hypotension due to rupture of segmental vessels may have further exacerbated intracranial hypotension caused by CSF leak, contributing to the subdural hemorrhage.

## 4. Conclusion

We conclude that intracranial hypotension was the most likely cause of subdural hemorrhage in our patient.
